# Facts and Fiction: The Impact of Hypothermia on Molecular Mechanisms following Major Challenge

**DOI:** 10.1155/2012/762840

**Published:** 2012-03-13

**Authors:** Michael Frink, Sascha Flohé, Martijn van Griensven, Philipp Mommsen, Frank Hildebrand

**Affiliations:** ^1^Trauma Department, Hannover Medical School, Carl-Neuberg-Straße 1, 30625 Hannover, Germany; ^2^Department of Trauma and Hand Surgery, Heinrich Heine University Hospital, Moorenstraße 5, 40225 Düsseldorf, Germany; ^3^Department for Experimental Trauma Surgery, Technical University of Munich, Ismaningerstraße 22, 81675 München, Germany

## Abstract

Numerous multiple trauma and surgical patients suffer from accidental hypothermia. While induced hypothermia is commonly used in elective cardiac surgery due to its protective effects, accidental hypothermia is associated with increased posttraumatic complications and even mortality in severely injured patients. This paper focuses on protective molecular mechanisms of hypothermia on apoptosis and the posttraumatic immune response. Although information regarding severe trauma is limited, there is evidence that induced hypothermia may have beneficial effects on the posttraumatic immune response as well as apoptosis in animal studies and certain clinical situations. However, more profound knowledge of mechanisms is necessary before randomized clinical trials in trauma patients can be initiated.

## 1. Introduction

A great number of patients with major injuries [1–3] suffer from accidental hypothermia ranging from 12 to 66% [4, 5]. In the current literature, multiple classifications using different definitions for hypothermia are described. In most classifications, hypothermia is defined as a core temperature below 35°C. Since an increase of mortality has been demonstrated below a core temperature of 34°C in patients with multiple injuries, this temperature threshold seems to be critical in trauma patients and therefore a modified definition is reasonable [6]. Thereby, the extent of hypothermia correlates with the overall injury severity and is increased by pelvic or abdominal surgery [7]; furthermore, hypothermic polytrauma patients suffer from an increased incidence of posttraumatic complications [2, 4, 8–10] as well as a an increased mortality [2, 8, 11].

Depending on its origin, three entities of hypothermia are known: endogenous, induced, and accidental hypothermia.

Endogenous hypothermia results from a metabolic dysfunction with a decreased heat production (e.g., hypothyreodism, hypoglycaemia, hypoadrenalism) or a disturbed thermoregulation (e.g., intracranial tumor, degenerative neurologic disorders).

Accidental hypothermia is characterized by an unintentional decrease of the core temperature due to exposure to a cold environment without a thermoregulative dysfunction [12]. This can be aggravated by therapeutic interventions. The infusion of cold fluids as, for example, 2 l of crystalloids (18°) decreases core temperatures about 0.6°C [13]. In addition, a reduced oxygen supply with an anaerobic state decreases the amount of adenosine trisphosphate (ATP) and subsequent heat production. Furthermore, the application of anaesthetics and skeletal muscle relaxants prevents shivering and vasoconstriction and therefore advances heat loss.

While the first two entities do not play a major role after trauma, accidental hypothermia is common in trauma patients as well as patients undergoing major surgery [4, 5]. In contrast to accidental hypothermia that needs to be addressed in the treatment of severely injured patients due to its detrimental effects, induced hypothermia is commonly used, that is, in elective cardiac surgery [14]. In addition there exists a strong recommendation for the induction of hypothermia after cardiopulmonary reanimation, and there exists a growing body of evidence that suggests the application of hypothermia after blunt brain injury. Some articles were published regarding the influence of hypothermia in context of elective surgery but also on the posttraumatic immune response in animal models. However, an overview about the influence of hypothermia on the humeral and cellular immune response with special focus on apoptosis is missing. This paper outlines the molecular mechanisms by which hypothermia influences apoptosis as well as the immune response following severe trauma and major surgery. 

## 2. Apoptosis

In general, cell death following hemorrhage and ischemia occurs either as necrosis of affected cells or as a complex process of programmed cell death called apoptosis. In contrast, necrosis represents the premature death of cells caused by external factors, that is, trauma, infections, or exposure to toxins.

Apoptosis is associated with a cascade of enzymatic reactions in which proteolytic caspase enzymes play a major role. Apoptosis could be initiated either in an extrinsic or intrinsic way [60]. The extrinsic way is characterized by the interaction of ligands (e.g., TNF-*α*) with “death receptors” on the cell surface (e.g., CD95) activating the enzymatic cascade by caspase-8 [61]. The intrinsic activation of apoptosis is triggered by tumor suppressing factors (e.g., p53) resulting in an increased expression of proapoptotic factors of the bcl-2 family (e.g., bax, bad) and an increased mitochondrial release of cytochrome-c [62]. Binding Apaf-1 (apoptotic protease activating factor) cytochrome-c activates the apoptotic cascade via caspase-9 [63, 64]. Finally, programmed cell death is mediated by “effector caspases,” especially caspase-3, in both ways of apoptotic activation. Besides the proteins of the bcl-2 family (antiapoptotic bcl-2; proapoptotic bax and bad), the apoptotic process is regulated by Mitogen-activated protein kinases (MAP-kinases) like extracellular-signal-regulated kinase 1/2 (ERK 1/2), cJun N-terminal protein kinase 1/2 (JNK 1/2), and p38 MAP-kinase. Additionally, other pathways involving Phosphatidylinositol-3 kinases (PI3K) generating phosphatidylinositol-3,4,5-trisphosphate (PI-3,4,5-P(3)) and other phospholipids interacting with Akt are known to play an important role in regulating cell survival [65]. Furthermore, besides kinases the transcription factor NF-kappaB regulates cellular apoptotic programs [66].

In various studies, hypothermia was shown to prevent additional tissue injury by interrupting both, the intrinsic, and extrinsic apoptotic pathway [15, 67–70]. Interestingly, hypothermia seems to affect the very early steps in the apoptotic process including inhibition of activation of caspase enzymes, preserving mitochondrial function and decreased overload of excitatory transmitters. In contrast, apoptosis occurs relatively late following tissue challenge but it was shown that the process continues for up to 3 days [70–72]. Due to the delay of the apoptotic process, modulation of the apoptotic cascade could serve as a therapeutic target in early stages of polytrauma management after the initial resuscitation process in which the patient is stabilized with the aim to prevent additional damage. In this context, it is of special importance that the rate of apoptosis in neutrophils is dramatically decreased in multiple trauma patients [73].

### 2.1. In Vitro Studies

Cultured hepatocytes showed suppressed FAS-mediated apoptosis detected by a decreased mitochondrial damage following moderate hypothermia. Besides an attenuated cytochrome-c release, hypothermia suppressed the activation of caspase-7 and -9 [74]. This data suggests potential organ protective effects of hypothermia regarding apoptosis, which were confirmed in various animal models. On the other hand, murine neutrophils revealed a reduced spontaneous and TNF*α*-induced apoptosis under mild hypothermia of 35°C [75]. This fact could result in a prolonged exposure to activated neutrophils after trauma resulting in secondary organ damage.

### 2.2. Experimental Animal and Clinical Studies

Profound hypothermia was not only shown to preserve Akt in cardiomyocytes and inhibit caspase-3 activation but also activate antiapoptotic proteins such as bcl-2 in an experimental model of hemorrhagic shock [19] and ischemic insult [20]. In ischemia/reperfusion injury, hypothermia reversed activation of apoptosis stimulating fragment (FAS)/caspase-8, the increase of bax (an proapoptotic protein), and decrease of bcl-2 in endothelial cells [16]. This was accompanied by an inhibition of JNK 1/2 activation via MKP-1 induction [16]. Following ischemia and reperfusion, isolated cardiomyocytes showed increased Phospho-Akt levels associated with attenuation of reactive oxygen species production, which was blocked by Akt but not cGMP inhibition [76]. Additionally, hypothermia was associated with downregulation of the TNF receptor (TNFR)1 and its proapoptotic ligand FAS in rat cerebral cortices following a moderate fluid percussion model of traumatic brain injury [77].

Most of the clinical studies regarding the influence of hypothermia on apoptosis are limited to ischemic injuries following cardiac arrest or brain ischemia [78, 79].

In summary, there is clear evidence that hypothermia reduces ischemic neuronal apoptosis in global cerebral ischemia as a result of attenuated p53 expression and increased bcl-2 release [78]. Information regarding multiple injuries is not available to date. The effects of hypothermia on apoptosis are summarized in Table 1.

## 3. Immune System

### 3.1. Humoral Inflammatory Response

Immune response following major surgery or trauma consists of a complex set of pro- and anti-inflammatory reactions in order to restore homeostasis. The balance or imbalance of the pro- and anti-inflammatory immune response in part influences the clinical course. Whereas predominance of the pro-inflammatory response leads to the Systemic Inflammatory Response Syndrome (SIRS), the anti-inflammatory reaction also named as compensatory anti-inflammatory response syndrome (CARS) might result in immune suppression with an enhanced risk of infectious complications. SIRS as well as immune suppression plays a decisive role in the development of sepsis and the Multiple Organ Dysfunction Syndrome (MODS) after trauma. Cytokines, released from various cell types including immunocompetent and intrinsic cells, regulate the specific and unspecific immune response. These mediators are detectable in the peripheral blood and several compartments like the lung and the liver. They serve not only as a marker of injury severity or outcome predictors but also as a tool for decision-making regarding timing of elective surgery during the clinical course [80, 81]. The most important cytokines in this regard include TNF-*α*, IL-1*β*, IL-6, IL-8, and IL-10 (Table 2). As another essential step of the systemic immune response, chemotactic cytokines, so-called chemokines (IL-8, MCP-1, MIP-1*α*, or MIP-1*β*) mediate neutrophil infiltration into the affected tissue [82]. Thereby, extravasation of neutrophils is mediated by different adhesion molecules [83]. The initial neutrophil-endothelial interaction, so-called rolling, is mediated by members of the selectin family of adhesion molecules. Integrins (CD11/CD18) and immunoglobulin superfamily receptors (ICAM-1, VCAM-1, ELAM-1) are important for the following firm adhesion and cell diapedesis [84, 85]. In various experimental as well as clinical studies, an effect of hypothermia on the inflammatory response by altering the expression of pro- and anti-inflammatory cytokines, chemokines and adhesion molecules has been shown [38, 86–88].

In brain injury, cytokines can have neuroprotective as well as neurotoxic properties. There is profound evidence that an inadequate or disproportionate posttraumatic immune response not only increases the risk for brain cell injury but also the extent of damage [89–95]. In this process, the IL-1 family plays a pivotal role. Elevated levels of IL-1 as well as an increased expression of IL-1 mRNA were detected following experimental brain injury in rodents, respectively [96–100]. While IL-1 does not cause brain damage itself, injection of IL-1 increased cell death following various brain damage models [101–103]. The hypothesis that IL-1 increases brain damage is supported by experiments in which an IL-1 antagonist prevented cell death in experimental brain injury [102–105]. Similar results were observed following treatment with an IL-1 converting enzyme (ICE) inhibitor in cerebral ischemia [106]. Thus, modulation of cytokine release by hypothermia may serve as a therapeutic approach following major injury.

#### 3.1.1. In Vitro Studies

Peripheral blood mononuclear cells stimulated with lipopolysaccharide (LPS) from healthy volunteers showed decreased TNF-*α* release, while release of IL-1 and IL-6 was delayed when incubated at 33°C as compared to incubation at 37°C [86]. In a similar study with human macrophages, early secretion of TNF-*α* and IL-6 was blunted and in human monocytes early IL-6 and IL-1*β* secretion was decreased [107]. Additionally, a shift towards anti-inflammatory cytokines was detected in microglia cells following LPS treatment [108]. In contrast, hypothermia of 33°C raised the levels of IL-1*β*, IL-6, and TNF-*α* produced by monocytes from healthy volunteers stimulated with LPS [109]. This controversial findings suggest diverse effects of hypothermia on different cell types.

#### 3.1.2. Experimental Animal Models

In a rat model of acute hemorrhage, Gundersen et al. evaluated the effect of hypothermia on immune response and corresponding organ damage. Moderate hypothermia had an organ protective effect in liver and kidney, which was associated with a decreased release of IL-6 as well as a reduction of reactive oxygen species [30]. In contrast, mild hypothermia did not affect systemic levels of IL-1, IL-6, and IL-10, while serum TNF-*α* levels were even increased following hemorrhagic shock suggesting different responses of cytokines or their respective sources [110].

In a study using a swine model of uncontrolled lethal hemorrhage, the authors were able to detect a decreased pro-inflammatory (IL-6) and an increased anti-inflammatory (IL-10) immune response following profound hypothermia. Furthermore, the potentially protective chaperone heat shock protein-70 (HSP 70) was preserved. The authors, therefore, concluded a beneficial modulation of the immune system due to hypothermia in this hemorrhage model [111].

Only a few publications investigated the anti-inflammatory effects of hypothermia in a combined trauma-hemorrhage setting. In a two-hit model consisting of a femoral fracture and hemorrhage, systemic IL-10 levels were elevated following mild hypothermia [44] confirming results from other experimental studies [35, 112, 113]. The increased anti-inflammatory response induced by hypothermia was also associated with a conversion from Th-1 to Th-2 cytokine pattern [35].

In a nonbacterial-driven sepsis model using intraperitoneal lipopolysaccharide injection, hypothermia also induced elevated plasma IL-10 levels [114].

The effects of hypothermia on the release of inflammatory cytokines are presented in Table 3.

#### 3.1.3. Clinical Studies

Experimental studies of traumatic brain injury, in which hypothermia decreased systemic cytokine levels, were confirmed by a clinical study showing a suppression in cytokine release associated with an improved outcome [38, 88]. In cerebral trauma patients, hypothermia of 32-33°C suppressed systemic IL-6 levels, which was associated with an increased Glasgow outcome scale 6 months postinjury as compared to patients treated under normothermic conditions [38].

### 3.2. Adhesion Molecules and Chemokines

The effects of hypothermia on chemokine levels and the expression of adhesion molecules were investigated in experimental as well as clinical studies (Tables 4 and 5).

#### 3.2.1. In Vitro Studies

In vitro studies of human umbilical vein endothelial cells showed decreased MCP-1 as well as IL-8 levels under hypothermic conditions [40]. In contrast, no effect of hypothermia on ICAM-1 expression in human cerebral endothelial cells was shown under stress conditions [41]. In cultured human umbilical vein cells, hypothermia inhibited the transcription and expression but not the induction of E-selectin [49].

#### 3.2.2. Experimental Animal Models and Clinical Studies

In a model of cerebral ischemia and reperfusion, mild hypothermia reduced local expression of MCP-1 [42]. The same finding could be confirmed in a murine multiple trauma model [44]. In another cerebral ischemia model, intraischemic as well as delayed hypothermia decreased ICAM-1 expression as well as intracerebral neutrophil infiltration [51].

In pediatric cardiopulmonary bypass (CPB) surgery, hypothermia reduced systemic levels of the chemokine regulated on activation normal T cell expressed and secreted (RANTES) and MCP-1 [45]. In contrast, no effect of hypothermia on MCP-1 levels was detected in a model of cardiac arrest in rats [43].

In transient focal cerebral ischemia, mild hypothermia reduced ICAM-1 expression, which was associated with reduced neutrophil and monocyte infiltration [50].

In contrast to experimental studies, circulating adhesion molecules were not altered by hypothermia in aortocoronary artery bypass grafting [54]. The effects of hypothermia on adhesion molecules are presented in Table 5.

In summary, there is evidence that induced hypothermia decreases pro-inflammatory cytokines as well as chemokines and adhesion molecules. Besides this, an increased anti-inflammatory cytokine response is observed in various trauma models. However, information of human studies is spare.

### 3.3. Cellular Immune Response

#### 3.3.1. Experimental Studies

Hypothermia influences the cellular immune response, which was especially studied following brain injury. In various animal models, neutrophil and macrophage function was attenuated leading to a decreased extent of secondary brain injury and infarction size [115]. Furthermore, posttraumatic hypothermia decreased early and prolonged accumulation of neutrophils and myeloperoxidase activity suggesting hypothermia as a potential mechanism to modulate outcome [116]. These findings confirmed an earlier study of Toyoda et al. showing a decreased neutrophil infiltration following intraischemic hypothermia in a focal ischemia reperfusion injury [55], which was also shown after delayed hypothermia in another cerebral ischemia model [51].

Additionally, posttraumatic hypothermia reduced neutrophil accumulation on the injury site at 24 h in a model of spinal cord injury [57].

Although most studies were conducted in traumatic brain injury, similar findings were shown in other organs. Already in the 1980s, it was shown that hypothermia reduced local neutrophil infiltration in an experimental pleuritis model, while the number of circulating neutrophils was not affected [117]. Other studies suggest a hypothermia-induced decrease of circulating neutrophils after soft-tissue injury in piglets [118], supporting an older study with prolonged hypothermia of 29°C in pigs [119]. The reduced infiltration can be explained by a decrease of adhesion molecules due to hypothermia. However, phagocytosis of opsonised bacteria is even increased at a lower temperature suggesting a temperature-dependent activation of neutrophils [48].

Following major injuries, infiltrated neutrophils release proteolytic enzymes as well as free radicals causing tissue damage which may subsequently lead to organ dysfunction and failure. Hypothermia was able to reduce the amount of free radicals in ischemic brain injury. In contrast, hypothermia did not affect the formation of free radicals in a rat model of hemorrhagic shock [120–122]. The reduced number of free radicals is of great benefit since the capacity of antioxidative mechanisms is limited. Interestingly, the prevention of free radical production is linearly linked to the temperature [123]. Most of free radicals following brain injury are synthesized by nitric oxide synthase and by deregulated mitochondrial electron transporters [124–127]. Thus, it was speculated that the prevention of release or synthesis of free radicals may be induced by preserved mitochondrial function [128]. Interestingly, mitochondrial function plays also a pivotal role in the development of apoptosis through inhibition of the caspase cascade activation. The essential role of the nitric oxide synthase was supported by another experiment, in which attenuation of acute lung injury by induced hypothermia following hemorrhagic shock was associated with less myeloperoxidase activity and decreased gene expression of iNOS. Furthermore, gene expression of heat shock protein (HSP-72), a molecular chaperone known to exert protective effects in ischemia-reperfusion injury, was detected in hypothermic but not in normothermic rats [29].

In contrast to these results, another study using a model of pressure-controlled hemorrhagic shock revealed no differences in serum-free 8-isoprostane (a marker of lipid peroxidation by free radicals) between the two groups at either baseline or resuscitation time 1 hr [120]. In a forebrain ischemia and recirculation model, hypothermia prevented production of hydroxyl radicals following hyperthermia [129]. In another study, postischemic leukotriene production a well as edema development was reduced 2 h but not 10 min following reperfusion [130]. The effects of hypothermia on the respiratory burst are summarized in Table 6.

#### 3.3.2. Clinical Studies

Controlled mild hypothermia had no effect on the number of circulating T lymphocytes in patients with severe brain injury [131]. A clinical study including infants and children with severe traumatic brain injury showed a preserved antioxidant reserve in cerebrospinal fluid, suggesting an attenuation of oxidative stress following hypothermia in severe brain injury [128].

## 4. Functional Parameters

### 4.1. Experimental Studies

Less neuron damage was detected at a temperature of 34°C following brain ischemia [132] confirming another study of thoracic aortic ischemia-reperfusion injury in which hypothermia prevented and delayed paralysis by preserving cells of the central nervous system [133]. In another study, a beneficial long-term effect of mild (35°C) and moderate (32°C) hypothermia was detected after spinal cord ischemia and reperfusion until 28 days following the injury [134].

In a rat model of ischemia/reperfusion of the lower extremity, local hypothermia protected skeletal muscle from capillary leakage, which was prevented after treatment with heme oxygenase and nitric oxide synthase inhibitors [135].

Hypothermia not only prevents damage on site of injury but also distant organ damage. In mesenteric ischemia-reperfusion injury, remote lung injury was prevented as measured by leukocyte trafficking, alveolar hemorrhage, and increased BAL protein and wet/dry ratios [136]. These results confirmed another study using a hepatic model of ischemia/reperfusion injury, in which hypothermia reduced the associated lung injury [137].

In a rat hemorrhagic shock model, mild hypothermia (34°C) improved survival associated with modulation of the immune response [110].

### 4.2. Clinical Studies

Clinical studies are mainly focused on patients with traumatic brain injury. In general, there is a gap between experimental studies and clinical experience. In a multicenter study of 392 patients with severe head injuries, hypothermia of 33°C did not influence outcome. In 5 meta-analyses, conflicting results regarding outcome were shown [138–142]. Only 2 of them [141, 142] described a marginal benefit regarding mortality and neurological outcome. Patients with elevated intracranial pressure seem to be the cohort with the most benefit of hypothermia [141]. Regarding these controversial results, in current guidelines hypothermia is recommended as a level III treatment option [143].

## 5. Cooling Rewarming Procedure

### 5.1. Experimental Studies

Only few experimental studies investigated the effect of different cooling and rewarming strategies.

Hypothermia at time of occlusion decreased infarct size in a myocardial ischemia-reperfusion injury supporting the results showing an increased cellular tolerance to ischemia under hypothermic conditions [144]. These results suggest starting hypothermia as soon as the hemodynamic and hemostaseological parameters were stabilized. In contrast, there is consistent evidence that rapid rewarming reverses the beneficial effects of induced hypothermia in traumatic brain injury [145]. Furthermore, gradual rewarming improved survival and attenuated remote acute lung injury after intestinal ischemia and reperfusion injury as compared to speed rewarming [146]. The mechanism behind this phenomenon is only partly understood. Considering the current literature, it seems likely that abrogation of the beneficial effects is associated with ATP depletion, energy failure, and consecutive mitochondrial dysfunction [145]. To date, detailed knowledge about exact mechanisms in different rewarming strategies is lacking.

### 5.2. Clinical Studies

The European Resuscitation Council recommends early hypothermia (32–34°C) for 12–24 hours following cardiac arrest and a slow rewarming procedure with 0.25–0.5°C per hour avoiding hyperthermia [147]. In a clinical study in 57 hypothermic patients, continuous arteriovenous rewarming resulted in less fluid requirement and less mortality as compared to standard rewarming procedures [11].

In summary, there is only minor understanding of the rewarming process but slow rewarming is recommended based on mostly experimental studies.

## 6. Perspective

Before induced hypothermia can be introduced in the clinical management of patients several limitations of the presented studies need to be considered. A potential limitation of all studies in which hypothermia is induced by a cardiopulmonary bypass machine is the fact that increase of cytokines may be due to the bypass procedure itself [111]. While numerous experimental as well as clinical studies regarding cardiac surgery, brain injury, or cardiac arrest are available information regarding hypothermia following major injuries is spare. Since there are still divergent results in experimental studies that are mostly limited to traumatic brain injury, the mechanisms by which hypothermia influences the posttraumatic immune response after multiple trauma need to be elucidated.

## Figures and Tables

**Table 1:** Effects of hypothermia on apoptosis.

Authors	Study design	Insult	Degree of hypothermia	Effects
Xu et al. [15]	In vitro study (mouse neurons)	Apoptosis using serum deprivation	33°C	caspase-3/8/9 ↓, cytochrome-c ↓, JNK ↓ (no effect on bcl-2, bax)
Yang et al. [16]	In vitro study (HUVEC*)	Ischemia using oxygen-glucose deprivation	33°C	caspase-3/8 ↓, bcl-2 ↑, bax ↓, JNK ↓
Pastuszko et al. [17]	Experimental study (piglet neurons)	Ischemia	33°C	caspase-3 ↓, bax ↓ (no effect on bcl-2)
Sahin et al. [18]	Experimental study (rat neurons)	Ischemia	34°C	caspase-3/9 ↓, bcl-2 ↑, bax ↓
Shuja et al. [19]	Experimental study (rat cardiac tissue)	Hemorrhage	15°C	bcl-2 ↑
Xiong et al. [20]	Experimental study (rat neurons)	Ischemia	32-33°C	caspase-3 ↓, bcl-2 ↑

*Human umbilical vein endothelial cells.

**Table 2:** Inflammatory cytokines.

	Induction	Synthesis	Effects
*TNF-*α**	hemorrhage, hypoxia,	macrophages, monocytes,	primary, proinflammatory cytokine
	ischemia, endotoxine	T-lymphocytes	IL-1*β* and IL-6 expression
			activation of coagulation
			prostaglandine E2, steroid release
*IL-1*β**	hemorrhage, hypoxia,	macrophages, monocytes,	primary, pro-inflammatory cytokine
	ischemia, endotoxine	endothelial cells	Synergistic effects with TNF-*α*
	C5a, TNF-*α*		
*IL-6*	LPS, IL-1*β*, TNF-*α*	T-/B-lymphocytes,	Secondary, pro-inflammatory cytokine
		monocytes, endothelial cells	induction of acute phase proteins (e.g., CRP, PCT)
			differentiation of lymphocytes
			activation of von NK-cells and neutrophils
			anti-inflammatory effect (IL-1*β*↓, TNF-*α*↓)
*IL-8*	IL-1*β*, TNF-*α*, bacteria,	T-lymphocytes, monocytes,	Secondary, pro-inflammatory cytokine
	LPS, hypoxia	neutrophils, endothelial cells	chemotactic effect on leukocytes
*IL-10*	TNF-*α*, IL-1*β*, endotoxine,	T-/B-lymphocytes,	anti-inflammatory cytokine
	LPS, prostaglandine E2	monocytes, macrophages	

**Table 3:** Effects of hypothermia on inflammatory cytokines.

Authors	Study design	Insult	Degree of hypothermia	Effects
Zheng et al. [21]	Experimental study (piglets)	Ischemia	18°C	TNF-*α*↓, IL-6 ↓
Sipos et al. [22]	Experimental study (pigs)	Ischemia	19/24/30°C	No effect TNF-*α*, IL-6, IL-10
Qing et al. [23]	Experimental study (pigs)	Ischemia	20/28°C	TNF-*α*↓, IL-10 ↑
Meybohm et al. [24]	Experimental study (pigs)	Ischemia	33°C	TNF-*α*↓, IL-1*β*↓, IL-6 ↓, IL-10 ↓
Su and Li [25]	Experimental study (pigs)	Ischemia	Not defined	TNF-*α*↓, IL-6 ↓
Lim et al. [26]	Experimental study (rats)	Inflammation	27°C	IL-1 *β*↓, IL-10 ↑
Fujimoto et al. [27]	Experimental study (rats)	Inflammation	32°C	IL-6 ↓, IL-10 ↑
Stewart et al. [28]	Experimental study (mice)	Inflammation	32°C	IL-6 ↑, IL-10 ↑; no effect on TNF-*α*, IL-1*β*
Kim et al. [29]	Experimental study (rats)	Hemorrhage	27–30°C	IL-6 ↓, IL-10 ↑
Gundersen et al. [30]	Experimental study (rats)	Hemorrhage	32.5–33°C	IL-6 ↓, no effect on TNF-*α*, IL-10
Beiser et al. [31]	Experimental study (mice)	Hemorrhage	33°C	IL-6 ↓
Wagner et al. [32]	Experimental study (mice)	Septic shock	27°C	IL-6 ↓
Vitarbo et al. [33]	Experimental study (rats)	Trauma (TBI)	33°C	TNF-*α*↓
Morita et al. [34]	Experimental study (rats)	Trauma (Lung)	34°C	TNF-*α*↓, IL-6 ↓, IL-10 ↑
Lee et al. [35]	Experimental study (rats)	Hypothermia (isolated)	30°C	IL-2 ↓, IL-10 ↑
Qayumi et al. [36]	Experimental study (pigs)	Lung transplantation	ex vivo preservation at 4°C	No effect on TNF-*α*, IL-2, IL-4, IL-10, Thromboxan
Shiozaki et al. [37]	Clinical study	Trauma (TBI)	34°C	No effect onTNF-*α*, IL-6, IL-10
Aibiki et al. [38]	Clinical study	Trauma (TBI)	32-33°C	IL-6 ↓

**Table 4:** Effects of hypothermia on chemokines.

Authors	Study design	Insult	Degree of hypothermia	Effects
Dalen et al. [39]	In vitro study (human neurons)	Ischemia using oxygen-glucose deprivation	33°C	No effect on IL-8, MCP-1
Diestel et al. [40]	In vitro study (HUVEC*)	Inflammation	17/32°C	IL-8 ↓, MCP-1 ↓
Sutcliffe et al. [41]	In vitro study (HCEC**)	Inflammation	32°C	IL-8 ↓
Zheng et al. [21]	Experimental study (piglets)	Ischemia	18°C	IL-8 ↓
Li et al. [42]	Experimental study (rats)	Ischemia	32-33°C	MCP-1 ↓
Callaway et al. [43]	Experimental study (rats)	Ischemia	33°C	No effect on MCP-1 and MIP-1*α*
Hildebrand et al. [44]	Experimental study (mice)	Hemorrhage	27–30/30–33/33–35°C	MCP-1 ↓
Eggum et al. [45]	Clinical study	Surgery	25/32°C	RANTES and MCP-1↓
Menasche et al. [46]	Clinical study	Surgery	~26°C	No effect on IL-8

*Human umbilical vein endothelial cells.

**Human cerebral endothelial cells.

**Table 5:** Effects of hypothermia on adhesion molecules.

Authors	Study design	Insult	Degree of hypothermia	Effects
Haddix et al. [47]	In vitro study (HUVEC*)	Inflammation	25°C	E-Selectin ↓
Sutcliffe et al. [41]	In vitro study (HCEC**)	Inflammation	32°C	No effect on ICAM-1, CD 18 (Integrin)
Frohlich et al. [48]	In vitro study (human leukocytes)	Inflammation	33/35°C	CD 11b (Mac-1) ↑, no effect on CD62L (L-Selectin)
Johnson et al. [49]	In vitro study (HUVEC*)	Hypothermia (isolated)	25°C	E-Selectin ↓
Meybohm et al. [24]	Experimental study (pigs)	Ischemia	33°C	ICAM-1 ↓
Callaway et al. [43]	Experimental study (rats)	Ischemia	33°C	No effect on ICAM-1
Wang et al. [50]	Experimental (rats)	Ischemia	33°C	ICAM-1 ↓
Deng et al. [51]	Experimental study (rats)	Ischemia/Inflammation	33°C	ICAM-1 ↓
Hanusch et al. [52]	Experimental study (rats)	Hypothermia (isolated)	Ex vivo storage after flushing lungs with cold fluid (4°C)	ICAM-1 ↓, VCAM-1 ↓, ELAM-1 ↓
Choi et al. [53]	Experimental study (rats)	Ischemia	33°C	ICAM-1 ↓
Menasche et al. [46]	Clinical study	Surgery	~26°C	No effect on P- und E-Selectin
Boldt et al. [54]	Clinical study	Surgery	27-28°C	No effect on ICAM-1, VCAM-1, ELAM-1

*Human umbilical vein endothelial cells.

**Human cerebral endothelial cells.

**Table 6:** Effects of hypothermia on proteins/molecules associated with the production of reactive oxygen species.

Authors	Study design	Insult	Degree of hypothermia	Effects
Toyoda et al. [55]	Experimental study (rats)	Ischemia	30°C	MPO ↓
Lim et al. [26]	Experimental study (rats)	Inflammation	27°C	MPO ↓
Kim et al. [29]	Experimental study (rats)	Hemorrhage	27–30°C	Malondialdehyde (TBARS)↓, MPO ↓, iNOS ↓, NO ↓
Duz et al. [56]	Experimental study (rats)	Trauma (spinal cord)	27–29°C	Malondialdehyde (TBARS) ↓
Chatzipanteli et al. [57]	Experimental study (rats)	Trauma (spinal cord)	32°C	MPO ↓
Grezzana Filho et al. [58]	Experimental study (rats)	Ischemia	26°C	Malondialdehyde (TBARS)↓,
Qayumi et al. [36]	Experimental study (pigs)	Lung transplantation	ex vivo preservation at 4°C hypothermia)	No effect on Superoxide-Dismutase, GR*, GP**
Hsu et al. [59]	Experimental study (rats)	Heatstroke	Perfusion with cold fluid (4°C)	Malondialdehyde (TBARS) ↓ Glutathione ↑, GP** ↑, GR* ↑, Superoxid-Dismutase ↑
Eggum et al. [45]	Clinical study	Surgery	25/32°C	No effect on MPO

*GR (Glutathione-Reduktase).

**GP (Glutathione-Peroxidase).

***GST (Glutathione-S-Transferase).
